# National survey of patient perspectives on cost discussions among recipients of copay assistance

**DOI:** 10.1093/oncolo/oyae148

**Published:** 2024-06-12

**Authors:** Anh B Lam, Ryan David Nipp, Jill S Hasler, Bonnie Y Hu, Greg J Zahner, Sarina Robbins, Stephanie B Wheeler, Erin K Tagai, Suzanne M Miller, Jeffrey M Peppercorn

**Affiliations:** Department of Medicine, University of Oklahoma Health Sciences Center, Oklahoma City, OK 73104, United States; Section of Hematology and Oncology, Department of Medicine, University of Oklahoma Health Sciences Center, Stephenson Cancer Center, Oklahoma City, OK 73104, United States; Fox Chase Cancer Center, Temple University Health System, Philadelphia, PA 19111, United States; Perelman School of Medicine, University of Pennsylvania, Philadelphia, PA 19104, United States; Section of Hematology & Oncology, Department of Medicine, Massachusetts General Hospital, Boston, MA 02114, United States; HealthWell Foundation, Germantown, MD 20874, United States; University of North Carolina at Chapel Hill, Chapel Hill, NC 27599, United States; Fox Chase Cancer Center, Temple University Health System, Philadelphia, PA 19111, United States; Fox Chase Cancer Center, Temple University Health System, Philadelphia, PA 19111, United States; Section of Hematology & Oncology, Department of Medicine, Massachusetts General Hospital, Boston, MA 02114, United States

**Keywords:** financial toxicity, cancer, autoimmune disease, copay assistance programs, cost discussion

## Abstract

**Background:**

Individuals with cancer and other medical conditions often experience financial concerns from high costs-of-care and may utilize copay assistance programs (CAP). We sought to describe CAP recipients’ experiences/preferences for cost discussions with clinicians.

**Methods:**

We conducted a national, cross-sectional electronic-survey from 10/2022 to 11/2022 of CAP recipients with cancer or autoimmune conditions to assess patient perspectives on cost discussions. We used multivariable logistic regression models to explore associations of patient perspectives on cost discussions with patient characteristics and patient-reported outcomes (eg, financial toxicity, depression/anxiety, and health literacy).

**Results:**

Among 1,566 participants, 71% had cancer and 29% had autoimmune conditions. Although 62% of respondents desired cost discussions, only 32% reported discussions took place. Additionally, 52% of respondents wanted their doctor to consider out-of-pocket costs when deciding the best treatment, and 61% of respondents felt doctors should ensure patients can afford treatment prescribed. Participants with depression symptoms were more likely to want doctors to consider out-of-pocket costs (OR = 1.54, *P* = .005) and to believe doctors should ensure patients can afford treatment (OR = 1.60, *P* = .005). Those with severe financial toxicity were more likely to desire cost discussions (OR = 1.65, *P* < .001) and want doctors to consider out-of-pocket costs (OR = 1.52, *P* = .001). Participants with marginal/inadequate health literacy were more likely to desire cost discussions (OR = 1.37, *P* = .01) and believe doctors should ensure patients can afford treatment (OR = 1.30, *P* = .036).

**Conclusions:**

In this large sample of CAP recipients with cancer and autoimmune conditions, most reported a desire for cost discussions, but under one-third reported such discussions took place.

## Teaser

Patients with cancer and other medical conditions have financial concerns from high costs-of-care and may use copay assistance programs. This study sought to describe CAP recipients’ experiences/preferences for cost discussions with clinicians.

**Implications for practice**

Individuals with cancer and other medical conditions often experience financial toxicity and may seek help from copay assistance programs (CAP). We sought to describe CAP recipients’ experiences/preferences for cost discussions. In this national survey of CAP recipients with cancer or autoimmune conditions, most reported a desire for cost discussions, but under one-third reported that such discussions occurred. We also investigated factors associated with preferences for cost discussions and found unique associations with patients’ clinical/demographic factors, financial toxicity, depression symptoms, and health literacy. Findings provide novel insights regarding CAP recipients’ perspectives on cost discussions, which could inform future efforts targeting financial toxicity.

## Introduction

Patients with cancer and other chronic medical conditions (eg, autoimmune conditions) often experience financial concerns from growing costs-of-care, which can lead to financial toxicity.^1^ Financial toxicity, first coined in the setting of cancer but now recognized throughout medicine, refers to the financial distress resulting from healthcare costs that impacts patients as well as their family and caregivers’ quality of life, care delivery, and physical/psychological symptoms.^2-10^ Moreover, financial toxicity can result in skipped medications, bankruptcy, and may impact survival.^11,12^ Efforts are urgently needed to identify and address financial toxicity among patients with cancer and other chronic medical conditions.

Copay assistance programs (CAPs) have emerged as a potential strategy to help mitigate financial toxicity.^13-15^ CAPs represent a form of patient assistance programs (PAPs) typically sponsored by pharmaceutical companies or charitable foundations.^16,17^ These programs seek to provide financial assistance in different forms, including copay cards, reimbursement, or deductible coverage.^13,18,19^ The implementation of CAPs may generate significant savings for patients, particularly in oncology in setting of expensive cancer-directed therapies. However, by contributing to copay assistance, pharmaceutical companies may seek to shield patients from direct financial liability while maintaining high prices for third-party payors, thus maintaining high drug costs.^14,18,20,21^ Notably, CAPs are limited in addressing the totality of patients’ financial concerns with additional gaps contributing to financial toxicity, potentially including the lack of cost-of-care discussions, inability to identify sufficient/sustained financial resources, and insufficient support for navigating the healthcare system.^11,13,18,22^

Existing literature suggests that patient–clinician discussions about costs-of-care may lead to better medication adherence and lower out-of-pocket expenses.^22,23^ However, cost discussions rarely take place in routine clinical practice, and limited published data exist identifying patients’ perceptions and preferences for such discussions.^24-28^ Recipients of CAP grants represent a group that has required financial support to gain access to care, and we know little about their preferences and experiences related to discussing costs-of-care with their medical team(s).^29^ Thus, CAP grant recipients are uniquely poised to provide insight regarding their experience with financial toxicity so clinicians may better understand how to evaluate and address patients’ concerns related to costs-of-care, as well as their experiences and preferences for cost discussions.

In the current study, we sought to describe experiences with, and preferences for, cost discussions among a large national cohort of CAP grant recipients with a cancer diagnosis or autoimmune condition. Our study sample comprises of CAP grant recipients of HealthWell Foundation (HWF), a 501c3 charitable foundation that establishes disease-specific funds that cover high-cost medications for the given disease. In our study sample, we included patients receiving support for a drug treating cancer or an autoimmune disease. These two conditions represent large groups of individuals who may require high-cost medications for chronic, rather than episodic care. This group serves as a unique population because little is known about the perspectives of CAP grant recipients regarding their desire for and experiences with cost discussions and financial toxicity. Further, while financial toxicity and communication preferences in oncology have been previously studied, including another large group of patients with a chronic condition offers the opportunity to evaluate differences across populations. We aimed to describe CAP grant recipients’ desire to discuss costs, preference for their doctors to consider out-of-pocket costs, belief that doctors should ensure patients can afford the treatment prescribed, and whether their doctor had discussed costs with them. We also sought to explore patient characteristics associated with their perspectives on cost discussions.

## Methods

### Study design

We conducted a national, cross-sectional study of CAP grant recipients receiving assistance from HealthWell Foundation. An anonymous, self-administered electronic survey was piloted in August 2022 and then administered from October 2022 to November 2022. Four separate recruitment emails were sent to recipients with a link to the web-based REDCap survey, including an introduction email and 3 follow-up emails. The REDCap survey remained open for responses for 1 month. Eligible participants included individuals aged 18 years or older who received a copay assistance grant from HWF between January 2021 and July 2022 for their diagnosed cancer or autoimmune-related disease. Participant eligibility for HWF depends on multiple factors including, but not limited to, available disease funds for the specific disease at time of application, having a form of health insurance, medication type, and income. Potential CAP grant recipients of HWF are commonly identified via referrals from clinicians and patient-advocates, pharmacies, and drug manufacturers. Financial assistance from HWF covers prescription copays, health insurance premiums, deductibles, coinsurance, and behavioral health services. HWF receives funding from various sources, including individual and corporate donors, which include pharmaceutical companies. The Mass General Brigham Institutional Review Board reviewed this study and deemed it exempt.

### Demographics and health characteristics

We asked participants to self-report information about demographics and medical characteristics (eg, race, ethnicity, education, employment, geographic region, and disease type). For purposes of this analysis, disease type was categorized into autoimmune disorders, hematologic malignancy, metastatic solid tumor disease, and non-metastatic solid tumor disease. Race was categorized into White, Black, and all other races, and ethnicity was categorized into Hispanic and non-Hispanic. We organized education levels into less than college, college or more, and prefer not to answer. We organized employment status into employed, retired, and other. We organized annual household income into $20,000-$39,999, $40,000-$59,999, $60,000-$79,999, $80,000-$99,999, greater than $100,000, and less than and greater than $60,000. We asked respondents about their insurance coverage with the following options: Medicare, Medicare Advantage, private, Medicaid, Both Medicare and Medicaid, other (Tricare or other military insurance), and not sure (respondents could check all options that applied). Due to the high percentage of people with Medicare in our sample, we categorized these responses into Medicare Advantage, traditional Medicare, and Non-Medicare groups. We organized geographic regions as: Northeast, Midwest, West, and South. We used a self-reported version of the Charlson Comorbidity Index (CCI) to assess comorbidity scores.^30^ We asked respondents about each CCI condition (omitting the disease for which participants received copay assistance) and categorized scores into CCI score of 0, 1, 2, and 3 or more.

### Perspectives on cost discussions

Patients were asked to report their level of agreement with the following 4 statements: (1) “I wanted my doctor to discuss costs with me”; (2) “I wanted my doctors to consider my out-of-pocket costs when deciding on the best treatment for me”; (3) “Doctors should make sure patients can afford the treatments they are prescribing”; and (4) “My doctor discussed drug costs with me.” Response options were on a 5-point Likert-type scale from strongly disagree (1) to strongly agree (5). In efforts to identify characteristics of individuals who agreed with or perceived these statements to be true, we dichotomized responses into “agree” (agree, strongly agree) or “other” (disagree, strongly disagree, neither agree nor disagree).

### Patient-reported outcomes

**Figure 1.**
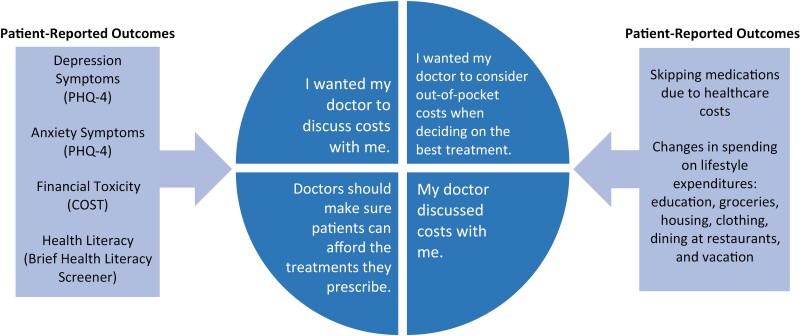
Patient-reported outcomes explored.

The patient-reported outcomes assessed included financial toxicity, depression and anxiety symptoms, and health literacy (Figure 1). To assess financial toxicity, we used the comprehensive score of financial toxicity (COST) tool, with severe financial toxicity defined by scores of 0-13.^31,32^ We used the Patient Health Questionnaire-4 (PHQ-4) to screen for anxiety and depression symptoms.^33^ This tool included 4 items: 2 items evaluating depression symptoms and 2 items evaluating anxiety symptoms.^33^ Higher scores indicated higher amounts of psychological distress.^33^ We used the Brief Health Literacy Screener to assess participants’ health literacy, using the 3 screening items that help detect inadequate health literacy.^34^ These items include: (1) “How often do you have someone help you read hospital materials?”; (2) “How confident are you filling out medical forms by yourself?”; and (3) “How often do you have problems learning about your medical condition because of difficulty understanding written information?” We categorized the health literacy levels as inadequate, marginal, and adequate. We further dichotomized these categories by combining inadequate and marginal groups, and comparing them to the adequate group. We asked if participants skipped medications due to healthcare costs. We also asked participants whether healthcare costs led them to spend less money on lifestyle expenditures, including education, groceries, housing, clothing, dining at restaurants, or vacation.

### Statistical analysis

We used descriptive statistics to describe patient characteristics overall and by diagnosis of cancer or autoimmune condition. We next built multivariable logistic regression models to describe the patient factors associated with patient perspectives and experiences for cost discussions. To build our regression models, we first fit univariable logistic regression models, in which each factor of interest was included as the only predictor in the model for each of the 4 statements. Factors that had a *P*-value < 0.10 in the univariable analysis were included in multivariable logistic regression models. Similarly, to explore associations among other patient-reported outcomes (ie, financial toxicity, depression and anxiety symptoms, and health literacy) with patient perspectives, we fit univariable logistic regression models, in which each patient-reported factor was included as the only predictor in the model. We then included each of these patient-reported factors in the multivariable logistic regression models to determine the association between each patient-reported factor and patient perspectives on cost discussions, controlling for demographic and clinical factors.

## Results

Among a national sample of 19,770 CAP grant recipients with cancer and autoimmune conditions, 1,566 responded to the survey (response rate 7.9%). Among these 1,566 responding participants, 71% (*n* = 1,108) reported receiving copay assistance for a cancer diagnosis and 29% (*n* = 458) for an autoimmune condition (Table 1). Participants had a median age of 71 years (IQR 66-75 years), and 51% were female. Among those with cancer, 67% had a hematologic malignancy, 18% had metastatic solid tumor, 4% had non-metastatic solid tumor, and 11% did not disclose their cancer type. The most common hematologic malignancies represented include multiple myeloma (*n* = 575), chronic lymphocytic leukemia (*n* = 73), and acute myeloid leukemia (*n* = 67). The most common cancer types represented include prostate cancer (*n* = 144), renal cell cancer (*n* = 76), and breast cancer (*n* = 32). Disease representation among respondents was similar in the invited survey population and reflected the specific funds the Foundation offered during the study period. For example, the majority of respondents had multiple myeloma (54%), which corresponds to 45% of all patients with cancer-related grants from HWF during the study period. Similarly, the majority of respondents with an autoimmune condition (76%) had multiple sclerosis, which reflect the disease supported by 70% of the Foundation’s autoimmune grants during this period. A majority of participants identified as White (83%) and non-Hispanic (97%). About half of participants had a Bachelor’s degree or greater (55%). Most participants were retired (79%), and 68% had household income less than $60,000. Nearly half of participants had traditional Medicare (52%) or Medicare Advantage (45%), reflecting the older age of our sample supported by charitable CAP. Compared to the general population, our cohort is more likely to be highly educated and more likely to have Medicare coverage.^35,36^ Geographic regions were represented as: South (36%), Midwest (23%), West (21%), and Northeast (20%).

**Table 1.** Patient demographics and characteristics.

Patient characteristic	Total *N* (%)	Cancer	Autoimmune
Mean age (range)	70.0 (22-94)	72.3 (41-92)	64.5 (22-94)
Median age (IQR)	71.0 (66-75)	72.0 (68-72)	66 (60-71)
<70	618 (43.0%)	345 (33.9%)	273 (65.2%)
≥70	820 (57.0%)	674 (66.1%)	146 (34.8%)
Gender	*n* = 362	*n* = 255	*n* = 107
Male	178 (49.2%)	154 (60.4%)	24 (22.4%)
Female	184 (50.8%)	101 (39.6%)	83 (77.6%)
Race	*n* = 1566	*n* = 1108	*n* = 458
White	1299 (91.4%)	912 (91.0%)	387 (92.1%)
Black	75 (5.3%)	58 (5.8%)	17 (4.0%)
All other races	48 (3.4%)	32 (3.2%)	16 (3.8%)
Ethnicity	*n* = 1455	*n* = 1034	*n* = 421
Hispanic	51 (3.5%)	35 (3.4%)	16 (3.8%)
Non-Hispanic	1404 (96.5%)	999 (96.6%)	405 (96.2%)
Education	*n* = 1460	*n* = 1037	*n* = 423
Less than Bachelor’s degree	584 (40.0%)	422 (40.7%)	162 (38.3%)
Bachelor’s degree or greater	801 (54.9%)	573 (55.3%)	228 (53.9%)
Prefer not to answer	75 (5.1%)	42 (4.1%)	33 (7.8%)
Employment	*n* = 1466	*n* = 1042	*n* = 424
Employed (full- or part-time)	120 (8.2%)	85 (8.2%)	35 (8.3%)
Retired	1156 (78.9%)	882 (84.6%)	274 (64.6%)
Unemployed	58 (4.0%)	34 (3.3%)	24 (5.7%)
Disability	91 (6.2%)	21 (2.0%)	70 (16.5%)
Other	41 (2.8%)	20 (1.9%)	21 (5.0%)
Household Income	*n* = 1296	*n* = 914	*n* = 382
< $20 000	112 (7.7%)	66 (6.4%)	46 (10.8%)
$20 000-39 999	432 (29.6%)	282 (27.2%)	150 (35.4%)
$40 000-59 999	347 (23.8%)	261 (25.2%)	86 (20.3%)
$60 000-79 999	242 (16.6%)	170 (16.4%)	72 (17.0%)
$80 000-99 999	107 (7.3%)	89 (8.6%)	18 (4.2%)
> $100 000	56 (3.8%)	46 (4.4%)	10 (2.4%)
Don’t know/prefer not to answer	164 (11.2%)	122 (11.8%)	42 (9.9%)
< $60 000	891 (68.8%)	609 (66.6%)	282 (73.8%)
≥ $60 000	405 (31.3%)	305 (33.4%)	100 (26.2%)
Insurance groups	*n* = 1559	*n* = 1103	*n* = 456
Traditional Medicare	805 (51.6%)	597 (54.1%)	208 (45.6%)
Medicare Advantage	699 (44.8%)	474 (43.0%)	225 (49.3%)
Non-Medicare	55 (3.5%)	32 (2.9%)	23 (5.0%)
Geographic region	*n* = 1333	*n* = 949	*n* = 384
Northeast	266 (20.0%)	188 (19.8%)	78 (20.3%)
Midwest	309 (23.2%)	205 (21.6%)	104 (27.1%)
West	277 (20.8%)	210 (22.1%)	67 (17.5%)
South	481 (36.1%)	346 (36.5%)	135 (35.2%)
Mean comorbidity score	*n* = 1566	*n* = 1108	*n* = 458
CCI 0	870 (55.6%)	622 (56.7%)	248 (54.1%)
CCI 1	302 (19.3%)	234 (21.1%)	68 (14.8%)
CCI 2	198 (12.6%)	128 (11.6%)	70 (15.3%)
CCI ≥ 3	196 (12.5%)	124 (11.2%)	72 (15.7%)

### Preferences for cost discussions

**Figure 2.**
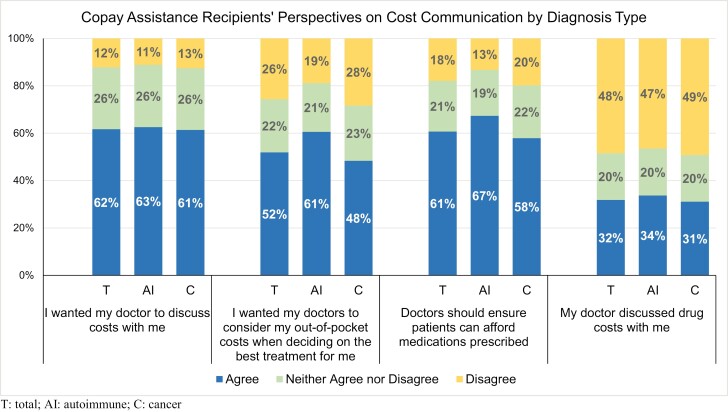
Copay assistance recipients’ perspectives on cost communication by diagnosis type. Abbreviations: AI, autoimmune; C, cancer; T, total.

While 62% (*n* = 880) of participants reported wanting their doctor to discuss costs, only 32% (*n* = 460) reported that cost discussions had taken place (Figure 2). Additionally, 52% (*n* = 734) wanted their doctor to consider out-of-pocket costs when deciding the best treatment, and 61% (*n* = 865) felt doctors should ensure patients can afford medications prescribed. Views were similar among patients with autoimmune conditions and those with cancer regarding the desire to discuss costs (63% vs 61%, *P* = .668) and cost discussions taking place (34% vs 31%, *P* = .323). Participants with an autoimmune condition were more likely to want doctors to consider out-of-pocket costs (60.6% vs 48.4%, *P* < .001) and believe doctors should ensure patients can afford medications prescribed (67% vs 58%, *P* < .001) when compared with participants with cancer.

### Patient factors associated with preferences for cost discussions

CAP grant recipients with a hematologic malignancy were less likely to want doctors to consider out-of-pocket costs (OR 0.58 [95% CI, 0.45-0.73]; Table 2), less likely to feel doctors should ensure patients can afford medications prescribed (OR 0.57 [95% CI, 0.42-0.78]), and less likely to report cost discussions took place (OR 0.67 [95% CI, 0.50-0.89]) compared to those with an autoimmune disorder. Participants with metastatic solid tumors were also less likely to want doctors to consider out-of-pocket costs (OR 0.65 [95% CI, 0.44-0.97]) and less likely to feel doctors should ensure patients can afford treatment (OR 0.60 [95% CI, 0.40-0.89]) compared to those with an autoimmune disorder. Participants who self-identified as Black race were less likely than those who identified as White to want their doctor to discuss costs (OR 0.56 [95% CI, 0.34-0.94]). Compared to participants who resided in the Northeast, participants who resided in the Midwest (OR 1.68 [95% CI, 1.15-2.47]) and in the West (OR 1.74 [95% CI, 1.16-2.58]) were more likely to report cost discussions took place.

**Table 2.** Univariable and multivariable analyses for patient demographics and characteristics and perspectives on cost discussions.

Characteristic	I want doctor to discuss costs with me				I wanted my doctors to consider my out-of-pocket costs when deciding on the best treatment for me				Doctors should ensure patients can afford medications prescribed				My doctor discussed drug costs with me			
	Univariable		Multivariable		Univariable		Multivariable		Univariable		Multivariable		Univariable		Multivariable	
	OR	95% CI	OR	95% CI	OR	95% CI	OR	95% CI	OR	95% CI	OR	95% CI	OR	95% CI	OR	95% CI
Current age	1.00	0.99-1.02			0.99	0.98-1.00	1.00	0.98-1.01	0.99	0.98-1.00			1.00	0.99-1.01		
<70	[ref]		[ref]		[ref]		[ref]		[ref]		[ref]		[ref]		[ref]	
≥70	1.18	0.95-1.46			0.93	0.75-1.15			0.92	0.74-1.14			1.06	0.84-1.32		
Gender																
Female	[ref]		[ref]		[ref]		[ref]		[ref]		[ref]		[ref]		[ref]	
Male	1.17	0.77-1.80			0.85	0.56-1.28			0.80	0.53-1.22			1.10	0.71-1.70		
Race																
White	[ref]		[ref]		[ref]		[ref]		[ref]		[ref]		[ref]		[ref]	
Black	0.59	0.37-0.94	0.56	0.34-0.94	1.01	0.63-1.61			0.90	0.56-1.44			0.57	0.32-0.99	0.73	0.40-1.33
All other races	1.46	0.78-2.75	1.39	0.70-2.75	1.10	0.62-1.96			1.42	0.77-2.65			1.12	0.61-2.05	0.78	0.37-1.65
Ethnicity																
Non-Hispanic	[ref]		[ref]		[ref]		[ref]		[ref]		[ref]		[ref]		[ref]	
Hispanic	0.53	0.30-0.93			0.82	0.47-1.43			1.14	0.63-2.05			0.79	0.42-1.48		
Education																
Less than college	[ref]		[ref]		[ref]		[ref]		[ref]		[ref]		[ref]		[ref]	
College or more	1.15	0.92-1.43	1.08	0.85-1.37	0.88	0.71-1.09	0.90	0.71-1.13	0.95	0.76-1.18	0.93	0.72-1.20	1.01	0.80-1.27	1.05	0.81-1.35
Prefer not to answer	0.47	0.29-0.77	0.45	0.26-0.77	0.64	0.39-1.05	0.46	0.27-0.79	0.66	0.41-1.08	0.59	0.34-1.04	0.57	0.32-1.01	0.57	0.30-1.08
Employment																
Employed	[ref]		[ref]		[ref]		[ref]		[ref]		[ref]		[ref]		[ref]	
Other	0.80	0.50-1.28			1.06	0.67-1.69			0.84	0.52-1.35			0.78	0.48-1.27		
Retired	0.88	0.60-1.31			0.76	0.52-1.11			0.85	0.57-1.25			0.88	0.59-1.30		
Household Income																
<$60 000	[ref]		[ref]		[ref]		[ref]		[ref]		[ref]		[ref]		[ref]	
≥ $60 000	1.15	0.90-1.47			0.87	0.69-1.10			0.81	0.64-1.04			0.90	0.70-1.16		
$20 000-39 999	0.99	0.64-1.52			1.36	0.89-2.07			0.83	0.53-1.29	0.90	0.54-1.50	0.97	0.63-1.51		
$40 000-59 999	0.82	0.53-1.27			1.17	0.76-1.80			0.71	0.45-1.12	0.79	0.47-1.34	0.91	0.58-1.43		
$60 000-79 999	0.94	0.59-1.49			1.10	0.70-1.73			0.72	0.45-1.15	0.84	0.49-1.45	0.75	0.46-1.21		
$80 000-99 999	1.48	0.84-2.63			1.10	0.64-1.88			0.57	0.33-0.99	0.68	0.36-1.29	1.04	0.60-1.83		
≥$100 000	0.95	0.49-1.85			0.93	0.49-1.78			0.55	0.28-1.06	0.51	0.24-1.09	1.00	0.51-1.97		
Don’t Know/Prefer not to answer	0.85	0.52-1.39			1.01	0.62-1.65			0.58	0.35-0.96	0.59	0.33-1.06	0.82	0.49-1.37		
Insurance Coverage																
Traditional Medicare	[ref]		[ref]		[ref]		[ref]		[ref]		[ref]		[ref]		[ref]	
Medicare Advantage	1.12	0.90-1.39			1.04	0.84-1.28			1.00	0.81-1.24	0.93	0.82-1.18	0.92	0.73-1.15		
Non-Medicare	0.88	0.80-1.53			0.87	0.50-1.51			2.33	1.21-4.49	1.98	0.97-4.05	0.71	0.38-1.33		
Geographic Region																
Northeast	[ref]		[ref]		[ref]		[ref]		[ref]		[ref]		[ref]		[ref]	
Midwest	1.11	0.79-1.55	1.09	0.77-1.54	1.12	0.81-1.56	0.93		1.00	0.71-1.42	0.98	0.67-1.42	1.73	1.21-2.50	1.68	1.15-2.47
West	1.42	1.00-2.02	1.28	0.89-1.84	0.99	0.71-1.39	1.05		0.71	0.50-1.00	0.73	0.50-1.06	1.69	1.17-2.46	1.74	1.16-2.58
South	1.15	0.84-1.56	1.13	0.82-1.55	1.10	0.81-1.49	0.92		0.85	0.62-1.16	0.83	0.59-1.16	1.42	1.01-2.00	1.42	0.99-2.05
Comorbidity Score																
CCI 0	[ref]		[ref]		[ref]		[ref]		[ref]		[ref]		[ref]		[ref]	
CCI 1	0.94	0.72-1.24			1.27	0.97-1.66	1.31	0.98-1.76	1.00	0.76-1.31			0.90	0.68-1.20	0.95	0.69-1.31
CCI 2	0.93	0.67-1.27			1.10	0.80-1.50	1.08	0.76-1.52	1.03	0.74-1.41			0.87	0.62-1.22	0.95	0.65-1.37
CCI 3+	1.06	0.76-1.47			1.36	0.99-1.88	1.35	0.95-1.91	1.27	0.91-1.76			0.71	0.50-1.01	0.68	0.45-1.03
Disease Type																
Autoimmune	[ref]		[ref]		[ref]		[ref]		[ref]		[ref]		[ref]		[ref]	
Metastatic (Met) Solid	1.27	0.89-1.81			0.68	0.49-0.96	0.65	0.44-0.97	0.60	0.42-0.84	0.60	0.40-0.89	1.19	0.84-1.69	1.13	0.78-1.65
Non-Met Solid	0.9	0.48-1.67			0.89	0.48-1.66	0.92	0.47-1.80	1.70	0.82-3.52	2.20	0.92-5.24	0.95	0.50-1.82	0.68	0.32-1.42
Hematologic	0.88	0.69-1.13			0.58	0.45-0.73	0.57	0.42-0.78	0.67	0.52-0.86	0.63	0.46-0.87	0.79	0.62-1.03	0.67	0.50-0.89
Years since diagnosis																
< 1	[ref]		[ref]		[ref]		[ref]		[ref]		[ref]		[ref]		[ref]	
1 to 4	0.90	0.53-1.53			1.25	0.75-2.10	1.31	0.75-2.29	1.31	0.79-2.17	1.26	0.70-2.26	0.75	0.44-1.26		
≥ 5	0.80	0.47-1.35			1.67	1.01-2.78	1.47	0.84-2.59	1.60	0.97-2.63	1.24	0.69-2.23	0.75	0.45-1.26		

### Patient-reported outcomes associated with preferences for cost discussions

Participants who screened positive for severe financial toxicity were more likely to want their doctors to discuss costs (OR 1.65 [95% CI, 1.27-2.14]; Table 3) and more likely to want their doctors to consider out-of-pocket costs when deciding on the best treatment for them (OR 1.52 [95% CI, 1.18-1.96]). Individuals with depression symptoms were more likely to want their doctors to consider out-of-pocket costs (OR 1.54 [95% CI, 1.14-2.08]) and to believe that doctors should ensure patients can afford their medications (OR 1.60 [95% CI, 1.15-2.21]) than those without depression symptoms. However, there was no difference in reported preferences nor experience of cost-discussions based on presence or absence of anxiety symptoms. When compared with participants with adequate health literacy, those with marginal or inadequate health literacy were more likely to want their doctors to discuss costs (OR 1.37 [95% CI, 1.08-1.73]) and more likely to believe doctors should ensure patients can afford medications (OR 1.30 [95% CI, 1.02-1.67]). Finally, participants who reported they skipped a medication due to cost in the past year were more likely to want their doctors to discuss costs with them (OR 1.54 [95% CI, 1.02-2.31]).

**Table 3.** Univariable and multivariable analyses of patient-reported financial toxicity, depression/anxiety symptoms, and health literacy on perspectives of cost discussions.

Characteristic	I wanted my doctor to discuss costs with me				I wanted my doctors to consider my out-of-pocket costs when deciding on the best treatment for me				Doctors should ensure patients can afford medications prescribed				My doctor discussed drug costs with me			
	Univariable		Multivariable		Univariable		Multivariable		Univariable		Multivariable		Univariable		Multivariable	
	OR	95% CI	OR	95% CI	OR	95% CI	OR	95% CI	OR	95% CI	OR	95% CI	OR	95% CI	OR	95% CI
Severe Financial Toxicity^a^	1.47	1.16-1.85	1.65	1.27-2.14	1.55	1.24-1.94	1.52	1.18-1.96	1.38	1.10-1.74	1.31	0.99-1.72	0.89	0.70-1.13	0.95	0.72-1.25
Anxiety^a^	1.24	0.96-1.60	1.31	0.99-1.74	1.21	0.95-1.55	1.14	0.87-1.50	1.34	1.04-1.72	1.29	0.96-1.73	1.05	0.81-1.35	1.15	0.86-1.54
Depression^a^	1.05	0.80-1.38	1.09	0.81-1.48	1.43	1.10-1.87	1.34	1.14-2.08	1.58	1.19-2.08	1.60	1.15-2.21	0.87	0.66-1.16	0.96	0.69-1.34
Inadequate/marginal Health Literacy ^b^	1.33	1.07-1.64	1.37	1.08-1.73	1.26	1.02-1.55	1.21	0.96-1.53	1.27	1.03-1.57	1.30	1.02-1.67	1.07	0.86-1.33	1.08	0.84-1.39
Skipped medication due to cost in past year^a^	1.45	1.00-2.11	1.54	1.02-2.31	1.58	1.10-2.25	1.31	0.87-1.98	1.55	1.07-2.25	1.30	0.84-2.01	1.15	0.8-1.65	1.17	0.77-1.76

^a^Reference group = no.

^b^Reference group = adequate health literacy.

### Lifestyle coping strategies and preferences for cost discussion

**Figure 3.**
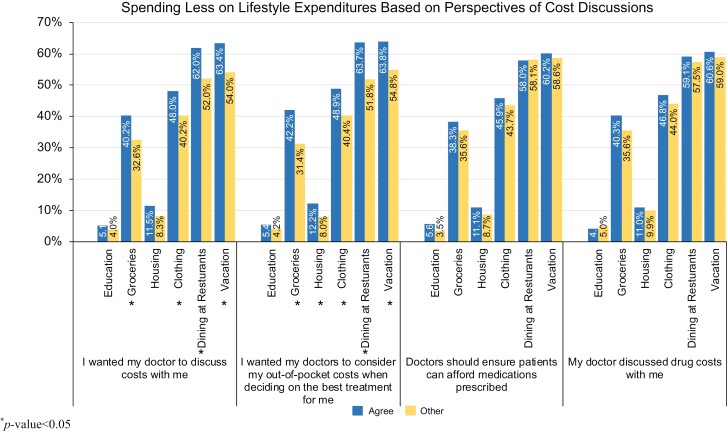
Spending less on lifestyle expenditures based on perspectives of cost discussions. ^*^P-value < .05.

Participants reported they reduced spending among several types of lifestyle expenditures (eg, groceries, clothing, and vacation) based on their preferences and experiences for cost discussions (Figure 3). Individuals who reported spending less money on groceries (40% vs 33%, *P* = .004), clothing (48% vs 40%, *P* = .004), dining at restaurants (62% vs 52%, *P* < .001), and vacation (63% vs 54%, *P* < .001) were more likely to want their doctors to discuss costs with them. Similarly, individuals who reported spending less on groceries (42% vs 31%, *P* < .001), clothing (49% vs 40%, *P* = .001), dining at restaurants (64% vs 52%, *P* < .001), vacation (64% vs 55%, *P* < .001), and housing (12% vs 8%, *P* = .008) were more likely to want their doctors to consider out-of-pocket costs when deciding the best treatment. There were no significant differences in reduced spending among those who did or did not prefer that doctors ensure patients can afford the medications prescribed nor whether or not the participants’ doctor discussed drug costs with them.

## Discussion

In this national survey of CAP grant recipients, we demonstrate that the majority of recipients reported a desire for cost discussions but also noted that these discussions rarely occurred. These findings align with prior literature in oncology, but our findings are particularly striking in this population of individuals with cancer and autoimmune disease who required financial assistance.^22,24,37,38^ In addition, we found that about half of CAP grant recipients preferred their physicians consider out-of-pocket costs when making treatment decisions, and just over half of respondents felt their physicians should ensure patients can afford the medications prescribed. We also investigated factors associated with preferences for cost discussions and found unique associations of preferences for cost discussions with patients’ clinical and demographic factors, financial toxicity, depression symptoms, and health literacy. Collectively, our findings provide novel insights regarding CAP grant recipients’ perspectives on cost discussions, which could help to inform future efforts to reduce financial toxicity among patients with cancer and other chronic medical conditions.

The findings of this study underscore that most participants reported a desire for cost discussions. Specifically, the majority (62%) of participants reported a desire for cost discussions with their doctors, which is higher than prior work and may be related to the unique study population of CAP grant recipients.^22,24,39,40^ In addition, the study sample may be biased by the fact that individuals willing to participate in a study addressing high costs-of-care may represent a population more likely to desire greater consideration of healthcare costs. However, our study cohort includes a large sample of participants with insurance coverage (particularly Medicare), suggesting that despite having insurance, individuals remain in need of additional financial assistance to afford medications. A more complete understanding of patients’ perspectives on cost discussions could be instrumental in: (1) identifying patients’ desires for cost discussions with their clinicians, (2) understanding how patient-clinician communication influences financial toxicity, and (3) developing interventions to reduce financial toxicity by implementing cost discussions in the clinical setting. This study supports the critical finding in the literature that patients often desire discussions about their financial concerns but rarely have the opportunity for such discussions in routine clinical practice.^22,24,26-28^ Thus, we need to better understand the barriers to such discussions in a clinic and develop interventions that help address this unmet need.

Notably, even within this population who required financial assistance, a sizeable minority of participants did not report a desire for cost discussions with their clinicians. This highlights the need for screening for financial burden and inquiring about patients’ desire to discuss costs, rather than assuming all patients want to discuss this topic. Additionally, this indicates the need for a personalized approach with patients as clinicians seek to understand patients’ interests in engaging in conversations regarding financial burdens and costs-of-care. Moreover, we found that only 32% of respondents reported their doctor had discussed costs with them. Without evidence-based methods to screen for financial distress in routine practice and implement meaningful patient-clinician cost discussions, clinicians and researchers miss a key opportunity to understand patients’ financial concerns and appropriately address barriers to reducing financial toxicity.^22,24,26-28,40^

To the best of our knowledge, the current study is one of the largest studies to ask CAP recipients about their perspectives regarding whether doctors should ensure patients can afford the treatment they prescribe. Specifically, 52% of participants wanted their physicians to consider out-of-pocket costs, and 61% felt that doctors should ensure patients can afford the treatment prescribed. Prior work suggests that patients want to discuss out-of-pocket costs and more studies are needed describing patient perspectives on out-of-pocket cost considerations during treatment planning.^22,39,40^ Asking physicians to consider out-of-pocket costs may seem inappropriate to some, as many clinicians believe patients should have access to effective treatment regardless of costs.^41^ Our study suggests that many, but not all, CAP grant recipients believe their out-of-pocket expenses should be taken into consideration, highlighting the need to identify patients who prefer cost considerations and methods to overcome barriers to communication about treatment affordability for patients with cancer and other chronic medical conditions.

Additionally, we identified patient characteristics associated with different perspectives on cost discussions. When compared to those with autoimmune conditions, those with hematologic malignancy and metastatic solid tumor reported a lower desire for physicians to consider out-of-pocket costs and ensure patients can afford medications. Those with hematologic malignancy also reported lower rates of cost discussions taking place, compared to those with autoimmune conditions. This warrants additional research to understand the mechanisms underlying this finding, and investigators should explore these patients’ treatment experiences and factors associated with reduced concern for cost. Our study also found an association between Black CAP grant recipients and a lower desire for cost discussions. The relatively low percentage of Black patients in this study suggests this finding should be viewed only as hypothesis-generating and explored further in future research.

We also found novel associations between CAP recipients’ perspectives on cost discussions with patient-reported financial toxicity, depression symptoms, and health literacy. We demonstrated an association between participants who met the criteria for severe financial toxicity and the desire for doctors to consider out-of-pocket expenses during treatment decision-making. This finding supports the notion that patients with higher financial concerns may be more interested in engaging in cost discussions with their physicians. We also found an association between depression symptoms and the desire for doctors to consider out-of-pocket expenses and the belief that doctors should ensure patients can afford their treatment. These key findings underscore the importance of addressing financial toxicity as financial toxicity may compound the psychosocial aspects of cancer and other serious illnesses.^42-45^ In addition, we found associations between low health literacy and desire for cost discussions and for doctors to ensure patients can afford their medications. This finding aligns with prior work suggesting that low health literacy often correlates with financial toxicity and supports the need to screen for and address financial toxicity in these patients.^46^ Lastly, participants’ preferences for cost discussions were associated with changes in spending habits and medication adherence. Individuals who have needed to change their healthcare and household spending have experienced the direct impact of financial toxicity, further demonstrating the critical need to address the high costs of cancer care.^29,47,48^

This study has several limitations that merit discussion. First, our study was limited to participants who were recipients of HealthWell Foundation copay assistance grant from a cancer- or autoimmune-related disease fund. Prevalence of disease among respondents reflects the disease-specific funds available during the study period. This was intended to be a select group of patients representing one end of the spectrum of financial burden, and the results are not broadly generalizable to patients who do not require assistance nor those served by manufacturer drug-specific PAPs. Second, while the number of respondents makes this one of the larger surveys of financial burdens and perspectives in the literature, the low response rate allows for potential bias, making estimates of the prevalence of the views reported less precise. We do not have detailed information on non-responders and are therefore unable to compare responders to non-responders, but based on the percentages of patients who received support from specific disease-based funds, it appears respondents were roughly representative of the surveyed population. We are not able to determine if patients with other sociodemographic differences or experiences were more or less likely to respond. What we can say with confidence is that a large number of patients express a desire for cost discussions in clinic that are not occurring, but the prevalence of these views and experiences may be higher or lower than reported here. It does, however, clearly demonstrate the diversity of views regarding cost discussions, highlighting the need to screen for this interest in clinical practice and individualized care.

Furthermore, we were not able to determine how many patients who were invited to participate opened and viewed the invitation, and therefore the true response rate among those considering the survey is unknown. Next, our insurance analyses are limited by our assumptions about our cohort: (1) they can accurately identify the type of insurance they have; (2) those who selected traditional Medicare and other kinds of insurance are referring to having supplemental insurance; and (3) those who selected only traditional Medicare likely also have additional insurance coverage. Additionally, the number of Black and Hispanic patients is remarkably under-represented in our survey sample. This serves as both a limitation in our study but also underscores a potential disparity in access to CAPs that warrants further investigation. Further, this was a cross-sectional survey; longitudinal data will be required to help understand whether and how these perceptions and experiences change over time.

In conclusion, this study, conducted among patients with a cancer diagnosis or autoimmune disease who were receiving CAP grants, describes a considerable gap between patients’ desires for discussions about prescription drug costs with their physicians and their experiences of such conversations. This gap was particularly notable for those with higher levels of financial toxicity and depression/anxiety symptoms and lower health literacy. This highlights the need for interventions to attend to the psychosocial/depressive symptoms of patients and ensure that interventions to address costs are targeted at appropriate literacy levels and cultural needs. This work confirms that this challenge is not unique to oncology and suggests that clinicians need to consider patients’ interests in addressing costs-of-care and be prepared to help address these costs to reduce financial burdens and ensure access to appropriate care.

## Data Availability

The data underlying this article will be shared on reasonable request to the corresponding author.
